# Preparation and Characterization of Ionic Conductive Poly(acrylic Acid)-Based Silicone Hydrogels for Smart Drug Delivery System

**DOI:** 10.3390/polym13030406

**Published:** 2021-01-27

**Authors:** Young-Ah Kim, Jin-Oh Jeong, Jong-Seok Park

**Affiliations:** Advanced Radiation Technology Institute, Korea Atomic Energy Research Institute, Jeongeup-si, Jeollabuk-do 56212, Korea; kya1214@kaeri.re.kr (Y.-A.K.); jojeong86@kaeri.re.kr (J.-O.J.)

**Keywords:** ionic conductive hydrogel, poly(acrylic acid), silicone, drug delivery, electron beam

## Abstract

In this study, we developed a smart drug delivery system that can efficiently deliver the required amounts of drugs using the excellent ion conductivity of poly(acrylic acid) (PAA) and an electrical stimulus. As a result of its having carboxyl groups, PAA hydrogel can be used in drug delivery patches to release drugs by ionic conductivity. However, PAA hydrogel has low durability and poor mechanical properties. The carboxyl group of PAA was combined with a siloxane group of silicone using electron-beam irradiation to easily form a crosslinked structure. The PAA–silicone hydrogel has excellent mechanical properties. Specifically, the tensile strength increased more than 3.5 times. In addition, we observed its cell compatibility using fluorescence staining and CCK-8 assays and found good cell viability. Finally, it was possible to control the drug delivery rate efficiently using the voltage applied to the ion-conductive hydrogel. As the voltage was increased to 3, 5, and 7 V, the amount of drug released was 53, 88, and 96%, respectively. These excellent properties of the PAA–silicone hydrogel can be used not only for whitening or anti-wrinkling cosmetics but also in medical drug-delivery systems.

## 1. Introduction

Smart drug delivery systems have recently been developed in which the drug delivery carriers respond to specific external stimuli provided to improve the drug-release efficiency. The human body has different physiological environments according to specific sites and disease states that change dynamically in response to specific stimuli [1,2,3]. Therefore, it is essential that the drug can be delivered to a target site and that the desired amount of the drug can be delivered to induce the desired drug effects. Accordingly, conductive drug delivery carriers are formed using stimulation-sensitive polymers or compounds that exhibit structural changes in response to external stimuli such as light, temperature, and electrical stimulation. Moreover, the release pattern can be controlled to suit the advantages of specific drug delivery [4,5,6,7]. In particular, a drug delivery carrier such as a hydrogel patch is a representative drug-release method for effective delivery of the drug into the skin using a micro-current without loss of the drug at the desired site or waste of time [8,9,10,11]. Drug delivery systems using electrical stimulation have the advantage that drugs can be delivered deep into the skin by controlling the intensity and duration of the current. In addition, drug delivery effects can be exhibited that are from tens to hundreds of times better than with drug administration simply applied to the skin without any external irritation [8,12,13]. Thus, this method is a promising method by which it is possible to release drugs where it is difficult to penetrate the skin and as a route through which drugs can be more efficiently administered.

Hydrogels are hydrophilic polymers with three-dimensional networks that can be altered by various physical, chemical and electrical stimulations [14,15,16,17]. In addition, with processing, it is easy to transform various forms according to the hydrogel used and the intended applications. These have good biocompatibility due to their high moisture content and physicochemical similarity to an extracellular matrix (ECM) [18,19,20]. In particular, when preparing hydrogels, their porous structures can easily be controlled to load drugs, thus enabling their application as drug delivery systems. A variety of pharmaceutical applications involving such as mask packs and contact lenses are already used for skincare [16,17]. Hydrogels are widely prepared using biocompatible polymers (e.g., polyvinyl alcohol, PVA and poly(acrylic acid), PAA) for drug release through electrical stimulus [21,22].

PAA hydrogels are widely used in drug delivery patches because hydrogels have excellent swelling properties and adhesion due to hydrogen bonding and covalent bonding [23,24]. In addition, PAA hydrogels are non-toxic compounds that have the advantage of easy chemical modification. They also have anionic properties (due to having carboxyl groups) that can be used as an electrode pad for smart drug delivery system [25,26]. However, because the hydrogels prepared using only PAA have disadvantages (low durability and weak mechanical properties), these hydrogels are poorly able to endure life activities when a PAA hydrogel patch is attached to the skin of a human body [27,28,29].

To overcome these problems, in this study, PAA–silicone hydrogels were prepared using electron-beam irradiation to improve the durability and mechanical properties. Silicone is an intermediary (organic–inorganic) material composed of siloxane bonds (Si–O–Si) and is harmless to the human body. It has excellent mechanical properties and heat resistance and is widely utilized in cosmetic and medical materials (e.g., silicone oil, silicone compounds for blood separation, and packing for artificial kidneys) [30,31,32]. However, silicone has the disadvantage that it does not exhibit conductivity because ions are not free to move in it. Therefore, the focus of this study was to determine how silicone could be improved by overcoming disadvantages such as poor durability and weak mechanical properties. The aim was to mix PAA and silicone to prepare ion-conductive hydrogels, if possible.

In this study, we prepared a smart drug delivery system that can efficiently deliver the required amounts of drugs and which provides excellent mechanical properties and biocompatibility at the same time. The PAA–silicone hydrogels were prepared using electron-beam irradiation. The PAA–silicone hydrogel has excellent biocompatibility and mechanical properties, including better adhesive, compressive, and tensile strength. In addition, it is possible to control the drug delivery rate efficiently according to the voltage applied to the ion-conductive hydrogel. Therefore, it appeared that this PAA–silicone hydrogel could be used not only in whitening or anti-wrinkling cosmetics but also in medical drug-delivery systems.

## 2. Materials and Methods

### 2.1. Materials

Poly(acrylic acid) (PAA, Jurymer AC-10H, composition 19–21% PAA, 1% acrylic acid, and 79–81% water) was purchased from Nihon Junyaku Co., Ltd. (Nishishimbashi, Japan) and silicone (HA690A, polydimethylsiloxane) was obtained from TMB (Chilgok, Korea). Glycerin and platinum (Pt) were purchased from Sigma-Aldrich (St. Louis, MO, USA). Dulbecco’s modified Eagle’s medium (DMEM) and penicillin–streptomycin (PS) were purchased from Gibco (Grand Island, NY, USA). Fetal bovine serum (FBS) was obtained from Hyclone (Logan, UT, USA). The porcine skin (Micropig^®^ Franz cell membrane, 1.5 cm × 1.5 cm × 400 μm) was purchased from Medi Kinetics Co., Ltd. (Pyeongtaek, Korea). All reagents and solvents used in the experiments were used without further purification.

### 2.2. Preparation of PAA-Silicone Hydrogel by Electron Beam

**Figure 1:**
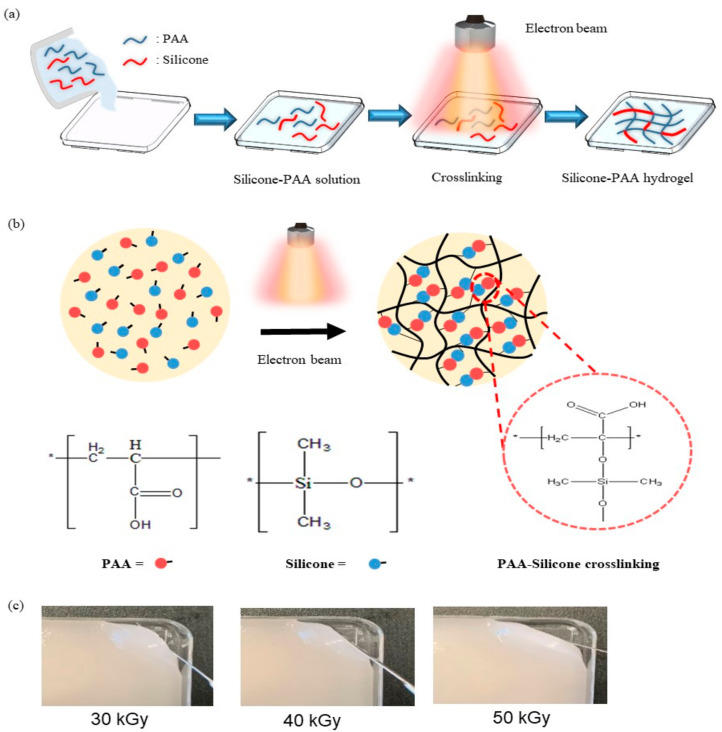
(**a**) Schematic illustration of the poly(acrylic acid) (PAA)–silicone hydrogels formed using electron-beam irradiation for crosslinking, (**b**) chemical mechanism of crosslinking induced using electron-beam irradiation (**c**) images of PAA–silicone hydrogels (Si 15) with different radiation doses (30, 40, and 50 kGy).

A solution of PAA and silicone was prepared with different concentrations of silicone (15, 20, and 25%) by mixing for 3 min and deforming for 5 min using a centrifugal mixer (ARE-310, THINKY Corporation, Tokyo, Japan). Next, 50 g of each of the prepared PAA–silicone solutions was put in a square dish (10 cm × 10 cm) to be exposed to electron-beam irradiation for crosslinking. They were exposed to electron-beam irradiation at 10 MeV (UELV-10-10S, at the Korea Atomic Energy Research Institute, Jeongup, Korea) with a radiation dose of 30, 40, or 50 kGy (10 kGy/cycle). The conditions of irradiation included a current of 3 mA and conveyor belt speed of 0.989 m/min, Hz. The feed ratios, schematic illustration, and optical images of the PAA–silicone hydrogel are shown in Table 1 and Figure 1.

### 2.3. Characterization of the PAA-Silicone Hydrogels

The chemical properties of the PAA–silicone hydrogels were confirmed using x-ray photoelectron spectroscopy (XPS, NEXSA, Thermo Fisher Scientific, Waltham, MA, USA) analysis. The XPS was equipped with a dual-beam low-energy electron/ion source at 0–5 eV by which to observe carbon (C1s), oxygen (O1s), and siloxane (Si2p). Then, the FTIR spectra were measured using attenuated total reflection, Fourier-transformed infrared spectroscopy (ATR-FTIR, Bruker TEMSOR 37, Bruker AXS. Inc., Karlsruhe, Germany). The samples were measured in absorbance mode from 500–4000 cm^−1^ with 64 scans and 4 cm^−1^ resolution.

The morphologies of the PAA–silicone hydrogels were investigated using scanning electron microscopy (SEM, TM3030, HITACHI, Tokyo, Japan) with an electron beam at 15 kV and a working distance of 8.1 mm. Prior to SEM imaging, the samples were covered with a layer of gold for 60 s using a sputter coater (Q150RS, QUORUM, Laughton, United Kingdom).

Dried PAA–silicone hydrogels were stirred in distilled water for 6 h. Then, the swollen PAA–silicone hydrogels were dried again in an oven at 40 °C for 2 days. The gel fraction of the PAA-silocone hydrogels were calculated using the following equation:

gel fraction (%) = (*W*_d_/*W*_i_) × 100 (1)

where *W*_d_ is the weight of the dried gel after extraction, and *W*_i_ is the initial weight of the dried gel before extraction.

The swelling ratio was measured using a dried PAA-silicone hydrogel sample after drying at 40 °C to record the initial weight. The PAA–silicone hydrogels were immersed in distilled water for different intervals at room temperature until an equilibrium state of swelling was reached. The swelling ratios of the PAA–silicone hydrogels were calculated from the following equation:

swelling ratio (%) = [(*W*_s_ − *W*_d_)/(*W*_d_)] × 100 (2)

where *W*_s_ and *W*_d_ are the weights of the swollen and dried samples, respectively.

To confirm the compressive strength of PAA–silicone hydrogels, the samples were prepared using an 8 mm biopsy punch for measurement with a texture analyzer (EZ-SX, Shimadzu, Japan). There was a distance of 10 mm between the sample and probe and a cross-head speed of 10 mm/min. The compressive strength of a sample was measured under 80% deformation. The pig membrane (25 mm × 25 mm) was used to determine the adhesive properties of the PAA–silicone hydrogels using TMS-Pro (Food Technology Co., Sterling, VA, USA). The samples were prepared with an 8 mm biopsy punch and then fixed to the probe (diameter 10 mm) using double-sided adhesive tape to fix the membrane to the surface of a stainless-steel plate. The samples on the probe were attached to the membrane so as to separate the probe from the membrane. The tensile strength of the PAA–silicone hydrogels were confirmed using a texture analyzer. The samples (1 cm wide and 0.5 cm thick) were prepared and then broken at a load of 10 kgf and speed of 10 mm/min.

The thermal property of the PAA–silicone hydrogels was measured using thermogravimetric analysis (TGA, TA Q600, TA Instruments, New Castle, DE, USA). Then, 15.0 mg of each sample was prepared and placed in a platinum pan. The analysis was performed at a heating rate of 10 °C/min from 30 to 700 °C under a flow of nitrogen gas at 100 µL/min.

The electrical conductivity of the PAA–silicone hydrogels was measured using a four-point probe method (Modysystems, Hanam, Korea) and linear-scan voltammetry from −1 to 1 V. The samples were prepared to size (1 cm × 1 cm), and their thickness measured using Vernier calipers (Mitutoyo, Japan). The resistance was analyzed using I–V (current–voltage) curves, and the electrical conductivity was calculated from the following equation:

conductivity (S/cm) = 1/(correction factor × thickness × resistance)(3)

### 2.4. In Vitro Cell Study

In vitro cell culture of the PAA–silicone hydrogels was performed according to ISO 10993-5 to provide an extracted solution prepared by immersing samples in DMEM at 37 °C for 24 h, and then the solution was passed through a 0.22 μm filter (Sartorius, Ltd., Epsom, UK) and diluted 2× using DMEM. NIH3T3 cells (seeding density of 1 × 10^4^ cells/well) were cultured in a 96-well plate in DMEM (containing 10% FBS and 1% PS) with 5% CO_2_ at 37 °C for 24 h. The culture medium was removed, and then the extracted solution was added to a plate and incubated with 5% CO_2_ at 37 °C for 24 h. To observe the adherent morphology of NIH3T3, on day 1 of the cell culture, staining of F-actin and nuclei was performed. Then, the extracted solution was removed and washed 3 times using PBS. Next, 3.7% paraformaldehyde was added and left for 15 min at RT for fixation. Permeabilization was conducted with cytoskeletal buffer solution (CSK buffer, 0.29 g NaCl, 0.5 mL Triton X-100, 0.06 g MgCl_2_, 10.30 g sucrose, 0.47 g HEPES buffer) for 10 min to block the cell using blocking buffer (1% bovine serum albumin in PBS) at 37 °C for 1 h. The cells were incubated in rhodamine–phalloidin (1:100), and Hoechst 33,258 (1:1000) dyes at 37 °Cfor 1 h. After washing with PBS, the fluorescence images of the stained samples were acquired using fluorescence microscopy (DMI3000B, Leica, Germany), and images were merged using Image J software (NIH, Bethesda, Maryland, MD, USA). On day 1, the cell viabilities of the PAA–silicone hydrogels were determined using the CCK-8 assay. The CCK-8 solution was prepared by mixing DMEM and CCK-8 in solution (9:1) and then incubating it with 5% CO_2_ at 37 °C for 2 h to measure cell viability using a microplate reader with absorbance at 450 nm.

### 2.5. Drug Release Test

An L-ascorbic acid solution was prepared so that the drug at different concentrations (1, 2, and 3%) was dissolved in DW at RT and then loaded into samples for 2 h at 100 RPM using a shaking incubator (SI-600R, Lab Companion). The drug-loaded samples were immersed in phosphate-buffered saline (PBS) and then incubated in a water bath (BS-21, JEOL TECH, Daejeon, Korea) at 37 °C and shaken at 100 RPM for 2 h. The released drug was acquired at predetermined time intervals of 10, 20, 30, 40, 50, 60, and 120 min to measure the optical density of the released drug using a microplate reader (PowerWave XS, Biotek, VT, USA) with absorbance at 319 nm.

### 2.6. In Vitro Drug Delivery

The cumulative drug (L-ascorbic acid) was determined using a Franz diffusion cell at different voltages (3, 5, and 7 V). L-ascorbic acid solution (i.e., 1, 2, and 3%) was loaded into samples for 2 h while shaking at 100 RPM. PBS (pH 7.4) was put in a receptor chamber with a volume of 8 mL in which the PBS was stirred (magnetic stirrer) until 37 °C. Then, the Franz cell membrane was placed on a water jacket. The PAA–silicone hydrogels were attached to the membrane to provide a flat-ground joint for fixation. The cumulative drug release was induced using a DC power supply (E3634A, Agilent Technologies, Santa Rosa, CA, USA) to conduct electrical stimulation via a negative charge on the sample and positive charge on the solution (3, 5, and 7 V with 0.016, 0.01, and 0.003 Ù, respectively). The released drug solution was acquired at predetermined time intervals (0, 5, 10, 15, 30, and 45 min) through the sampling port and then measured using a microplate reader (Cytation 5, BioTek, Seoul, Korea) with absorbance at 288 nm.

## 3. Results and Discussion

### 3.1. Characterization of the PAA-Silicone Hydrogels by Electron Beam

An electron beam has the advantage that it is able to cause reactions in various states (e.g., gas, liquid, and solid) and at room and low-temperature [33]. Because the ionization of atoms is caused, the chemical and physical properties of materials can easily be changed by breaking chemical bonds or causing crosslinking reactions. When manufacturing a hydrogel using electron-beam irradiation to induce crosslinking reactions, the physical and chemical properties of the hydrogel can be adjusted by controlling the radiation dose. In addition, hydrogels can be prepared by inducing polymerization that forms free-radicals without chemical additives such as initiators and crosslinking agents [29,34]. Thus, non-toxic hydrogels can be prepared without residual chemicals, and biocompatibility can be improved because an electron beam also has the advantages of crosslinking and sterilizing at the same time.

Figure S1 (Supplementary Material) shows the Chemical mechanism for the electron beam cross-linking of PAA-silicone. 

**Figure 2:**
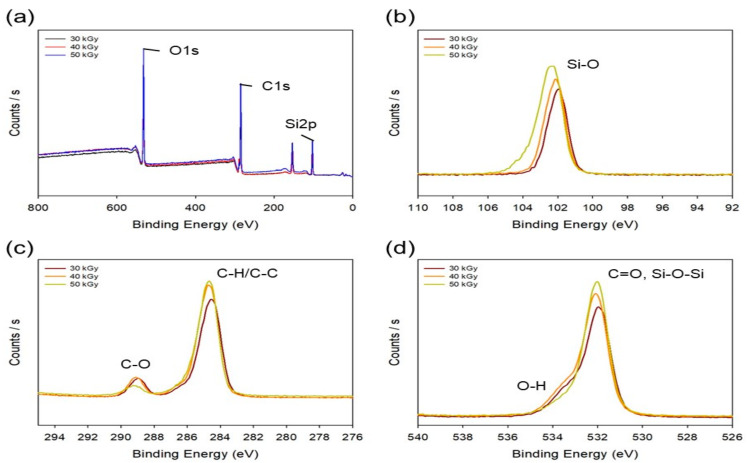
XPS spectra of the PAA–silicone hydrogels with silicone content of 25% and different radiation doses (30, 40, and 50 kGy): (**a**) all XPS spectra, (**b**) silicone (Si2p) only, (**c**) carbon (C1s) only, and (**d**) oxygen (O1s) only.

The chemical properties of Si 25 after different radiation doses (30, 40, and 50 kGy) were determined using XPS analysis. Figure 2a shows the overall XPS spectra and that the binding energy of silicone, carbon, and oxygen observed was at 100–106, 283–291, and 530–535 eV, respectively. As shown in Figure 2b for Si–O bonding, it was observed at 101.78, 101.91, and 102.13 eV. The peaks were shifted toward higher binding energies, and the peak intensity increased with the electron-beam dose. As expected for the mechanism in Figure S1b, it was believed that for Si–O, the radicals were formed in the structure of Si–O of silicon as well as PAA to expect that crosslinking reaction can be induced through the bonding of PAA and silicon. Figure 2c shows the binding energy at carbon. The binding energy of C–H and C–C was confirmed at 284.3 eV, and C–O was confirmed at 289.45 eV. In the case of C–C bonding, the intensity of the peak increased with an increase in the electron-beam irradiation dose. It is believed that the peak intensity of C–C was increased because the radicals in PAA formed by the electron-beam irradiation bind to each other to strengthen the C–C bond. In addition, the intensity of the C-O peak was increased due to the bonding of silicone and PAA. The intensity of the O-H peak was increased due to the bonding of silicone and PAA, whereas Si–O–Si bonding was strengthened due to silicone-to-silicone bonding, as shown in Figure 2d. Based on these results, we confirmed that PAA–PAA, PAA–silicone, and silicone–silicone bonds occurred simultaneously and that the reactions increased with the electron-beam irradiation dose. Nando’s team confirmed the mechanical properties by changing the chemical structure of silicone using electron-beam irradiation. When the electron beam was used, the mechanical properties (i.e., hardness and tensile modulus) of silicone were increased with the radiation dose. Through these results, it was possible to confirm crosslinking reactions in silicone induced by electron-beam irradiation [35]. This is based on our XPS results, which suggest crosslinking through the mechanism in Figure S1c.

**Figure 3:**
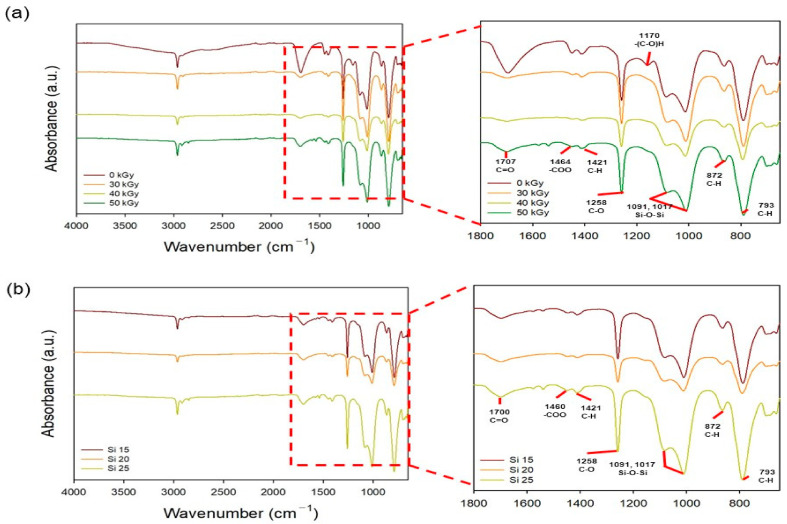
FTIR spectra of the PAA–silicone hydrogels: (**a**) silicone content of 25% with different radiation doses (30, 40, and 50 kGy) and (**b**) radiation dose at 50 kGy with different silicone content (15, 20, and 25%).

The chemical properties of the PAA–silicone hydrogels treated with different doses of electron-beam irradiation (and having different silicone content) were confirmed using ATR-FTIR. The CH_2_ peak was observed at 793 and 872 cm^−1^, and C=O and C–O peaks were observed at 1707 and 1258 cm^−1^, its main peak of PAA. In addition, Si–O–Si of the main peak of silicone was confirmed at 1091 and 1017 cm^−1^. As a result of confirming the chemical properties of Si 25 subjected to different electron-beam doses in Figure 3a, the Si–O–Si peak increases with the electron-beam dose. As in the results shown in Figure 2, we considered that the intensity of the Si–O–Si peak was increased because the bonding strength of Si–O–Si increased due to the formation of radicals in Si–O·and·Si of silicone by electron-beam irradiation. In particular, the intensity of the C–H peak (799 cm^−1^) decreased with an increase in the radiation dose. Because free radicals formed on the PAA chain (by H· or OH· radicals from water molecules) broke C–H bonds of PAA, the intensity of the C–H peak at 799 cm^−1^ decreased with increasing radiation dose. In addition, as shown in Figure 3b, the Si–O–Si peak at 1091 and 1017 cm^−1^ increased (with dose <50 kGy) with the increase in the radiation dose. This result shows that a large amount of silicone was joined due to an increase in the silicone content.

**Figure 4:**
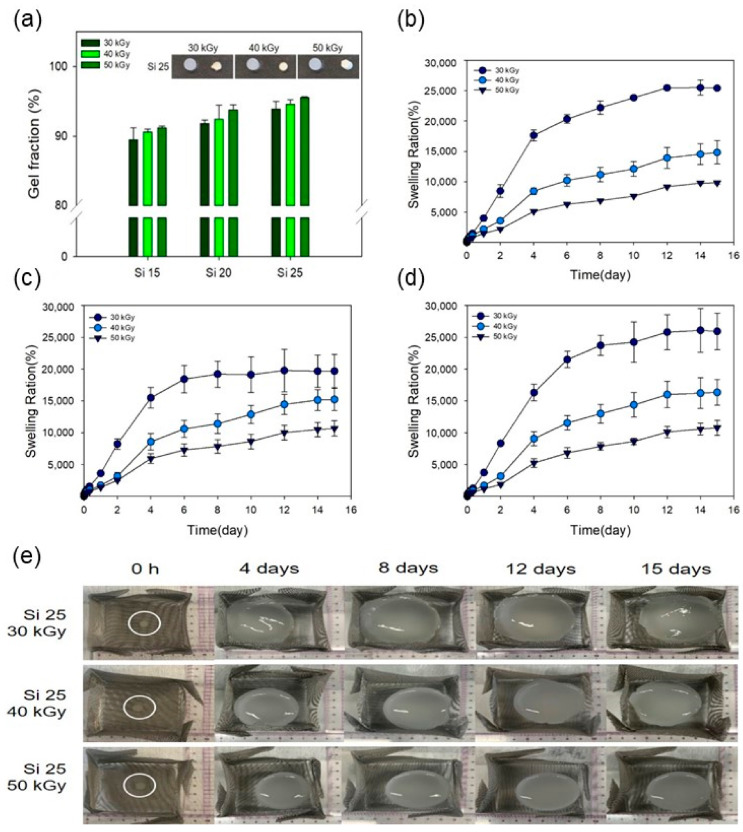
(**a**) Gel fraction of the PAA–silicone hydrogels, (**b**–**d**) swelling ratio of the PAA–silicone hydrogels with different silicone content (15, 20, and 25%), and (**e**) optical images of PAA–silicone hydrogels (PAA73Si25) with increased times.

The gel fractions of the PAA–silicone hydrogels were confirmed with different silicone contents and radiation doses, as shown in Figure 4a. After drying the PAA–silicone hydrogels, their weights were recorded and then the unreacted parts were removed using water to record the weight of the dried hydrogels and calculate the gel fraction. In the case of Si 15, the gel fraction rose with the increase in the radiation dose (for 30, 40, and 50 kGy, it was 89, 90, and 91%, respectively). These results show that the gel fraction increased due to an increase in the crosslinking within the PAA–silicone hydrogels as the electron-beam dose increased. In addition, the gelation rates of Si 20 were 92, 92, and 94%, respectively, and that the gelation rates of Si 25 were 94, 95, and 96%, respectively. As with the result for Si 15, it was confirmed that the gelation rate increased with an increase in the electron-beam irradiation dose. The gel fraction of Si 15 at 30 kGy (90%) was the lowest, whereas the gel fraction of Si 25 at 50 kGy (96%) was the highest. The reason that the gel fraction of Si 25 at 50 kGy was highest was due to more crosslinking reaction from the formation of a larger amount of free radicals according to greater silicone content and higher radiation dose. In addition, because the gel fraction was affected by the crosslinking density between polymer chains [36], the gel fraction increased with an increase in the radiation dose. Accordingly, we believed that Si 25 at 50 kGy would exhibit excellent mechanical properties due to the addition of silicone.

Figure 4b–d shows the swelling ratio of Si 15, Si 20, and Si 25 prepared using electron-beam irradiation. In addition, Figure 4e shows an optical image of the PAA–silicone hydrogel on different days (0, 4, 8, 12, and 15 d). Swelling is a phenomenon in which a substance (e.g., hydrogels and fibers) absorbs a solvent and increases its volume. Because a hydrogel is inevitable given contact with water during the preparation process, and because it has a hydrated network, a major part of its physical behavior is that it exhibits swelling behavior. When the hydrophilic-crosslinked polymer chain was in contact with water, the polymer chain interacted with the solvent molecules due to its relative thermodynamic affinity with water [37,38]. Therefore, the polymer chain was swollen in a hydrated state. In addition, to obtain the desired swelling ratio, it could be controlled through copolymerization with a hydrophobic or hydrophilic polymer. The swelling ratio is affected by the diffusion coefficient, surface properties, mobility, and mechanical properties throughout a hydrogel [39]. As shown in Figure 4b, in the case of Si 15 soaked for 10 d, the swelling ratio changed with increased radiation dose (at 30, 40, and 50 kGy, it was 23,832, 12,099, and 7646%, respectively). In addition, as shown in Figure 4c,d, for Si 20 it was 19,117, 12,874, and 8614%, respectively, while for Si 25 it was 24,252, 14,384, and 8616%, respectively. The swelling ratio increased according to the increase in the radiation dose. Figure 4e shows that the swelling of the PAA–silicone hydrogels reached equilibrium after 10 d. When the concentration of PAA was decreased, the swelling ratio of the PAA–silicone hydrogels decreased. Because PAA has a strong tendency to retain water, the swelling ratio of PAA hydrogels increases according to the PAA content [24]. This being so, the crosslinking density was increased by increasing the radiation dose. Moreover, the space containing water was decreased because the internal structure of the hydrogel became denser.

**Figure 5:**
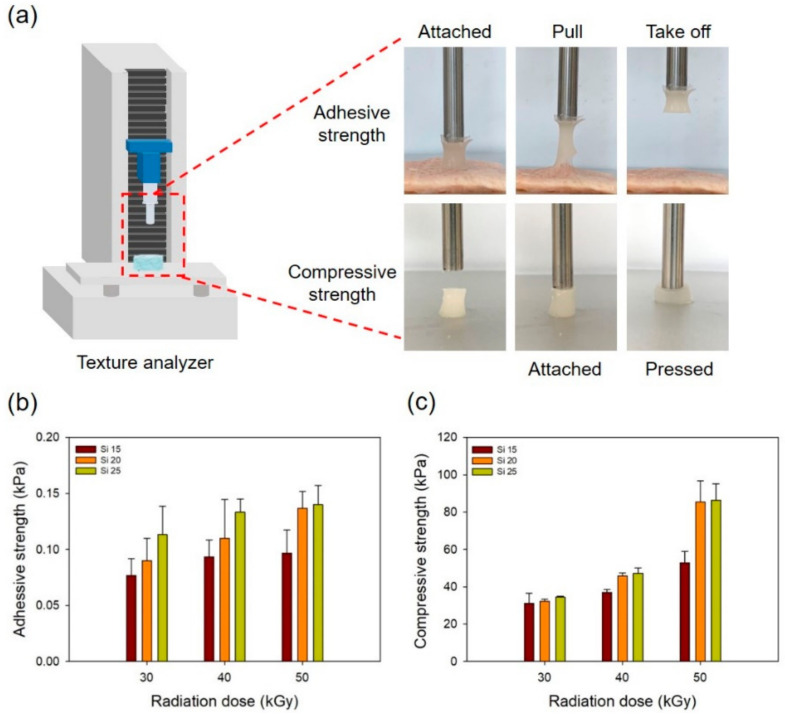
Adhesive and compressive strength of PAA–silicone hydrogels with different radiation doses and silicone content: (**a**) optical images, (**b**) adhesive strength, and (**c**) compressive strength.

Figure 5 shows the adhesive strength and compressive strength of the PAA–silicone hydrogels according to the silicone content and the radiation dose. Figure 5a includes a schematic diagram and optical images showing the adhesive and compressive strength of the PAA–silicone hydrogels. Each hydrogel was attached to the pigskin, and the adhesive strength was measured when the hydrogel was separated from the pigskin. As shown in Figure 5b, the adhesive strength of Si 15 increased with radiation dose (for 30, 40, and 50 kGy, it was 0.08, 0.09, and 0.10 kPa, respectively). With the same radiation doses, the adhesion strength of Si 20 was 0.09, 0.11, and 0.14 kPa, and of Si 25 was 0.11, 0.13, and 0.14 kPa. In our previous study, PAA hydrogel was prepared using electron-beam irradiation at 75 kGy with different PAA content (1 to 9%). The results of high gel fraction and low swelling ratio of the PAA hydrogel confirmed low adhesive strength with an increase of the PAA content due to decreased flexibility of the PAA chain by high crosslinking density [24]. However, in the case of PAA–silicone hydrogel, it was confirmed that the adhesive strength increased with the increase of the silicone content due to the adhesive properties of the silicone.

The compressive strength of the PAA–silicone hydrogels was measured by compressing the hydrogel to 80% deformation, as shown in optical images of Figure 5a. Figure 5c shows that the compressive strength of Si 15 increased with radiation dose (at 30, 40, and 50 kGy, it was 31, 37, and 53 kPa, respectively). At the same radiation doses, the compressive strength of Si 20 was 32, 46, and 85 kPa, and of Si 25 was 34, 47, and 86 kPa, respectively. These results showed that it was confirmed that high compressive strength was due to the higher crosslinking rate as the radiation dose and silicone content increased. In addition, the compressive strength observed for Si 20 and Si 25 was approximately twice as high as for Si 15 at a radiation dose of 50 kGy. It was shown that when the silicone content was >20%, the crosslinking density of the hydrogel was rapidly increased by electron-beam irradiation.

**Figure 6:**
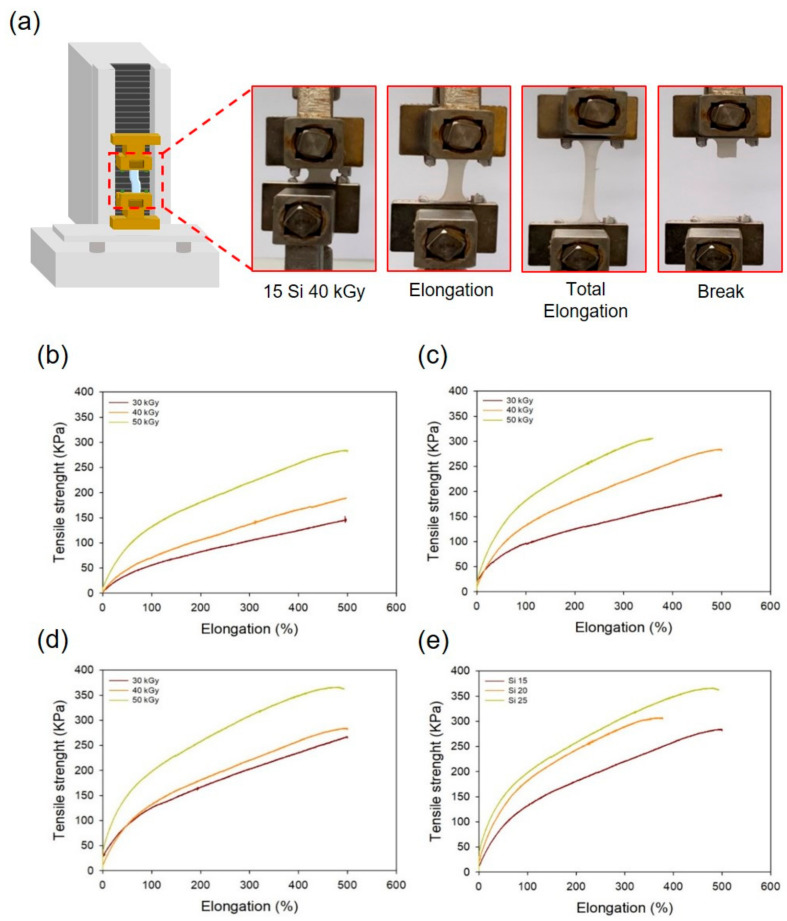
Tensile strength of the PAA–silicone hydrogels: (**a**) optical images, (**b**) silicone content of 15% with different radiation doses, (**c**) silicone content of 20% with different radiation doses, (**d**) silicone content of 25% with different radiation doses, and (**e**) radiation dose at 50 kGy with different silicone content (15, 20, and 25%).

When a hydrogel with weak mechanical properties is attached to the skin, it is easily torn, so it should not be used in a hydrogel patch. Therefore, a PAA hydrogel containing silicone was prepared due to improving its mechanical properties. This was determined by analysis of its tensile strength using a texture analyzer. Figure 6a shows a schematic diagram and optical images of the tensile strength of the PAA–silicone hydrogels. As shown in Figure 6b, the tensile strength of Si 15 (146, 189, and 282 kPa) increased with radiation dose. In Figure 6b,c, an increase in the tensile strength of Si 20 and Si 25 according to the increase of the radiation dose was also confirmed. The tensile strength of Si 20 was 192, 274, and 306 kPa, respectively. For Si 25, the tensile strength was 267, 283, and 362 kPa. As the silicone content increased, the tensile strength increased, and it is believed that the mechanical strength of the hydrogel was increased because it contained silicone.

### 3.2. In Vitro Cell Cytocompatibility

**Figure 7:**
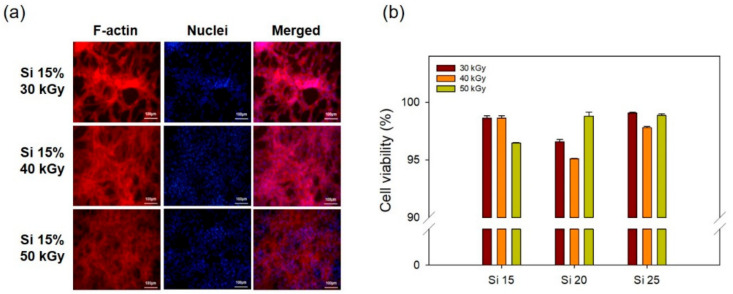
In vitro cell cytocompatibility of PAA–silicone hydrogels using fibroblast (NIH3T3) cells cultured for one day: (**a**) fluorescence images of samples stained with rhodamine–phalloidin and Hoechst (scale bar = 100 μm), and (**b**) cell viability using the CCK-8 assay.

To confirm the in vitro cell cytocompatibility of the PAA–silicone hydrogel, each hydrogel was reacted with only DMEM at 37 °C for 24 h. This was done to prepare the extracted solution for fluorescence staining (F-actin and nuclei) of fibroblast (NIH3T3) cells to indicate their viability. As shown in Figure 7a, when NIH3T3 cells were incubated for 24 h, the good cell morphology of Si 15 was confirmed by images of fluorescence stained cells (without change of cell adsorption or proliferation) with an increase of the radiation dose. In addition, Figure 7b shows cell viability using the CCK-8 assay. All hydrogels were confirmed to allow a high cell viability of 95%. This indicates the excellent biocompatibility of the PAA–silicone hydrogels. With the increase of radiation dose, cell viability of Si 15 was 99, 98, and 96, respectively. That of Si 20 was 96, 95, and 99; and that of Si 25 was 99, 98, and 99%, thereby confirming the high cell viability (>95%) of all the hydrogels. PAA is a good polymer for use in biomaterials because it allows good cell adhesion and proliferation due to its high hydrophilicity and excellent biocompatibility.

**Figure 8:**
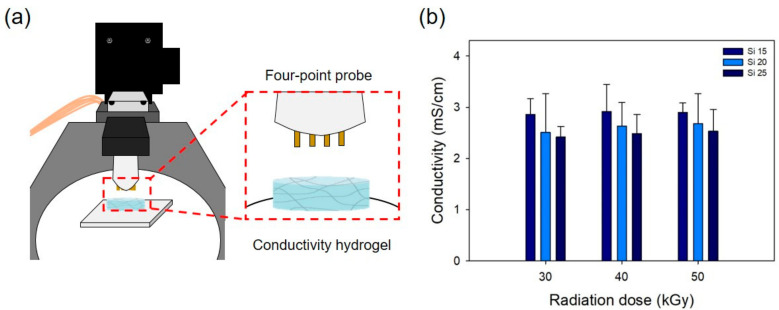
(**a**) Schematic diagrams of the conductivity test for the PAA–silicone hydrogels using the four-point probe method and (**b**) conductivity of the PAA–silicone hydrogels.

PAA hydrogels have anionic electrical properties due to having carboxyl groups. Figure 8 shows the electrical conductivity of a PAA–silicone hydrogel, and Figure 8a shows a schematic diagram of measurement using the four-point probe method. As shown in Figure 8b, with increase of the silicone content (15, 20, and 25%), the electrical conductivity was 2.86 ± 0.31, 2.92 ± 0.52, and 2.89 ± 0.18 mS/cm. Because the PAA content decreased with an increase in the silicone content, the anions of carboxyl groups decreased and thereby decreased the electrical conductivity. In addition, the electrical conductivity of hydrogel subjected to a radiation dose of 30 kGy was 2.51 ± 0.76, 2.64 ± 0.45, and 2.68 ± 0.59 mS/cm, and to a radiation dose of 40 kGy was 2.42 ± 0.20, 2.48 ± 0.36, and 2.53 ± 0.42 mS/cm. These electrical properties could be used to make drug release from PAA–silicone hydrogels more effective using electrical conductivity.

### 3.3. Drug Release Test

L-ascorbic acid is vitamin C. It is one of the most easily accessible vitamins and is contained in almost all foods. However, it is 20 times more effective to apply it to the skin than to consume it in food because much of this is lost [40,41]. In addition, it is a strong reducing agent and one of the essential components in the human body. It is involved in many activities such as activation of collagen synthesis, and scurvy occurs when L-ascorbic acid is deficient [42,43,44,45].

**Figure 9:**
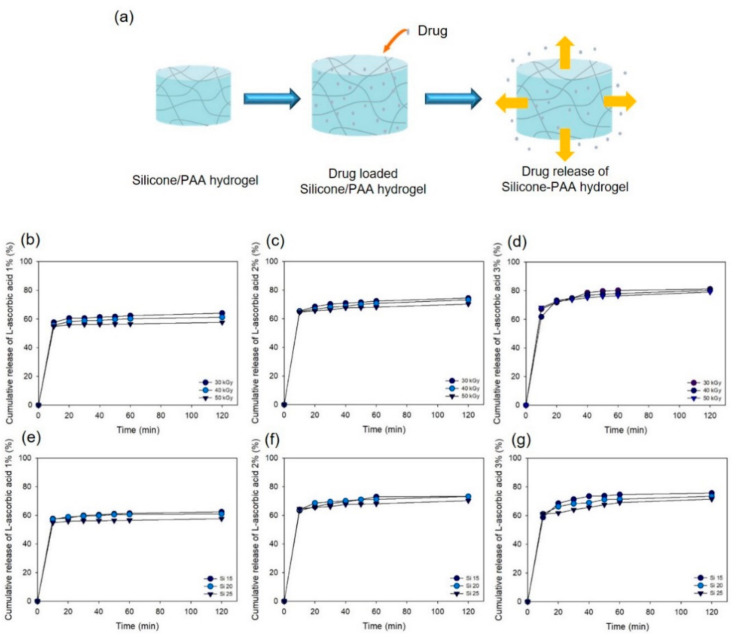
Cumulative release of L-ascorbic acid from PAA–silicone hydrogels over various time intervals (10, 20, 30, 40, 50, 60, and 120 min): (**a**) schematic diagrams of drug release, (**b**–**d**) hydrogel with silicone content 25%, different radiation doses, and varying drug content (1, 2, and 3%), and (**e**–**g**) radiation dose at 50 kGy with varying silicone content and drug content of 1, 2, and 3%.

After loading L-ascorbic acid into the PAA–silicone hydrogel for 2 h, the cumulative release was calculated after measurement using a microplate reader with absorbance at 319 nm. The schematic diagram in Figure 9a shows that the drug would be released from all sides in 37 °C PBS solution after loading the drug. High cumulative release of the drug could be predicted using the schematic diagram, and Figure 9b–d shows the amount of drug release at the drug concentration of Si 25 and with the increase of the radiation dose. The cumulative release of 1% L-ascorbic acid-loaded Si 25 with the increase of the radiation dose (30, 40, and 50 kGy) at 120 min was 64, 61, and 58%, respectively. In addition, the cumulative release of the drug loaded on the hydrogel with 2% Si 25 was 74, 73, and 70%, and with 3% Si 25 was 81, 80, and 79%, respectively. As the drug concentration was increased, it was confirmed that a larger amount of drug was released. When 1% and 3% drug-loaded hydrogel was compared at 120 min for Si 25 irradiated at 50 kGy, a difference in drug release of approximately 22% was confirmed. Figure 9e–g shows that the cumulative release of drug from Si 15, Si 20, and Si 25 according to increase of the drug content (i.e., 1, 2, and 3%) at the radiation dose of 50 kGy was confirmed. At 120 min, the cumulative release of 1% drug-loaded hydrogel (with silicone content of 15, 20, and 25%) was 63, 61, and 58%; for 2% drug-loaded hydrogel was 73, 73, and 70%; and for 3% drug-loaded hydrogel was 76, 74, and 72%, respectively. The initial pulse of drug release occurred at 20 min for the PAA–silicone hydrogel loaded with the drug. The change in the cumulative release of the drug was not significant in relation to increasing the silicone content. Based on these results, although a high amount of drug could be released in the body, the amount of drug delivered to the body from the skin surface by a hydrogel patch is not likely to be large.

### 3.4. In Vitro Drug Release Using Electrical Device

**Figure 10:**
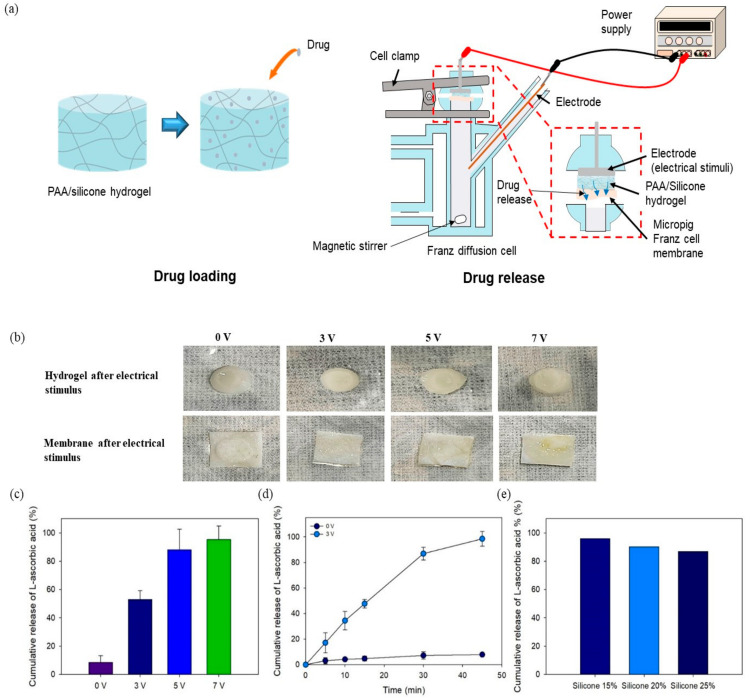
In vitro drug release from PAA–silicone hydrogels using a Franz diffusion cell at different voltages: (**a**) schematic illustration, (**b**) optical images of hydrogels and the Franz cell membrane, (**c**) cumulative release of drug at different voltages (3, 5, and 7 V) for 15 min, (**d**) cumulative release of drug at different voltages (0 and 3 V) for different intervals (5, 10, 15, 30, and 45 min), (**e**) cumulative release of drug from hydrogels with varying silicone content at a voltage of 3 V for 30 min.

Transdermal penetration of drugs is difficult because passing through the stratum corneum (the outermost layer of the skin) is difficult. Drug delivery using electrical stimulation enables the delivery efficiency to the skin through charged drugs [12,46]. In this experiment, effective skin penetration by the drug in relation to voltage was confirmed using a Franz cell. In addition, the porcine skin was used to Franz cell membrane. Figure 10a shows a schematic diagram of drug release assisted by electrical stimulation using Franz cells. A negative charge was applied to the drug-loaded hydrogel, and a positive charge was applied to the PBS solution. The interaction between the negative charge of PAA and the positive charge of PBS resulted in drug transfer to the solution exhibiting a positive charge. Figure 10b showed an optical image of the hydrogel and membrane according to the electric intensity after using a Franz cell. Although there was no change in the size of the hydrogel or of the membrane according to the increase of the voltage, the color of the membrane changed to slightly yellow when 7 V was applied. When the voltage was 7 V, the resistance was 0.003 R, and the current became 2333 A, which is ~2000 A higher than at 3 V (187.5 A) and 5 V (500 A). Figure 10c shows the cumulative drug release according to voltage increase for 15 min after loading 1% drug into Si 25 treated at 50 kGy. The amount of drug released by Si 25 treated at 50 kGy without voltage was 9%. As the voltage was increased to 3, 5, and 7 V, the amount of drug release was 53, 88, and 95%, respectively. When comparing the amount of drug release with Si 25 treated at 50 kGy without voltage with 3 V applied, there was a confirmed difference of ~45% or more. In addition, as shown in Figure 9b, the amount of drug release by Si 25 treated at 50 kGy was 58%. This was tested under conditions similar to those in the body. This result was similar (53%) to the result with 3 V of the applied voltage. In contrast, high drug release was confirmed at 5 V (88%) and 7 V (95%). Figure 10d shows the results of drug release according to the increase in time (5, 10, 15, 30, and 45 min). When the voltage was applied at 3 V, as shown in Figure 10c, the result was 53% for 15 min. As time passed, the results for 30 and 45 min were 98%. It was confirmed that there was more than twice the amount of previous drug release, and it was confirmed that almost all the drugs penetrated the skin within 45 min. In addition, Figure 10e shows the drug release for 30 min at 3 V according to the increase in the silicone content. As the silicone content increased, it was confirmed that the amount of drug release decreased. These results indicated that the PAA content was decreased by an increase of the silicone content, which decreased the negative charge. However, it was not lowered significantly, and the release of more than 80% of the drug was confirmed.

## 4. Conclusions

In this study, we prepared PAA–silicone hydrogels using electron-beam irradiation for a smart drug delivery system. This new system can efficiently deliver the required amounts of drugs and provides excellent mechanical properties and biocompatibility at the same time. The combination of PAA with silicone resulted in an increase in tensile strength of >3.5 times. In addition, tests of PAA–silicone hydrogels confirmed good cell viability (>95%). Finally, it was possible to control the efficiency (i.e., the drug delivery rate) by managing the voltage applied to the ion-conductive hydrogels. It was confirmed using a Franz cell that the amount of drug released from a drug-loaded PAA–silicone hydrogel was maximized when using an electrical stimulus. As the voltage increased (3, 5, and 7 V), the amount of drug released also increased. It was concluded that PAA–silicone hydrogels could be used not only in whitening or anti-wrinkling cosmetics but also in medical drug-delivery systems.

## Figures and Tables

**Table 1:** Feed ratios of the PAA–silicone hydrogels.

	PAA (%)	Silicone (%)	Glycerin (%)	Platinum (%)
Si 15	82.2	15	2	0.2
Si 20	77.8	20	2	0.2
Si 25	72.8	25	2	0.2

## Data Availability

The data presented in this study are available on request from corresponding author.

## Supplementary Materials

The following are available online at https://www.mdpi.com/2073-4360/13/3/406/s1. Figure S1: Chemical mechanism for the electron beam cross-linking of PAA-silicone: (**a**) PAA with PAA, (**b**) PAA with silicone, and (**c**) Silicone with silicone.
