# Supplementary material for: Preparation and Characterization of Ionic Conductive Poly(acrylic Acid)-Based Silicone Hydrogels for Smart Drug Delivery System
Source: Polymers (Basel). 2021 Jan 27;13(3):406. doi: 10.3390/polym13030406 (PMC7865612; doi:10.3390/polym13030406)
Supplement: Supplementary file 1 [file polymers-13-00406-s001.pdf]

Supplementary Material

Preparation and Characterization of Ionic Conductive Poly(acrylic Acid)-Based Silicone Hydrogels for Smart Drug Delivery System

Young-Ah Kim †, Jin-Oh Jeong † and Jong-Seok Park *

Advanced Radiation Technology Institute, Korea Atomic Energy Research Institute, Jeongeup-si, Jeollabuk-do, 56212, Korea; kya1214@kaeri.re.kr (Y.-A.K.); jojeong86@kaeri.re.kr (J.-O.J.)

* Correspondence: jspark75@kaeri.re.kr; Tel: +82-63-570-3067

† These two authors contributed equally to this work.

In this study, Silicone/PAA hydrogels were prepared using electron beams irradiation. Silicone can be complement for the disadvantages of poor durability and mechanical properties of PAA. Depending on the silicone content (*i.e.*, 15, 20, and 25%), PAA solution and silicone were mixed using centrifugal mixer and each solution (50 g) was placed on a square dish (10 x 10 cm), and a silicon/PAA hydrogel with a thickness of 1 cm was prepared. As shown in Figure S1, while the Silicone/PAA solution was exposed to electron beams, H· and OH· were formed from water molecules in the jurymer to form radicals in the C-H bond of PAA. The hydrogel was prepared from PAA radicals that induced a crosslinking reaction through covalent bonds to stabilize each other. Previously, we were confirmed about the chemical mechanism of induction of the PAA crosslinking reaction using radiation [23]. In addition, the radicals were formed in the structure of Si-O of silicon as well as PAA to expect that crosslinking reaction can be induced through the bonding of PAA and silicon. The silicone was expected to crosslinking through the covalent bonds between silicone and silicone. Generally, the one of the crosslinking method of silicone is heat hardening with catalyst at 200 °C for 4 h. However, since the crosslinking reaction using heat hardening has a disadvantage that have to react for a long time at a high temperature, radiation can be induced more easily and quickly.

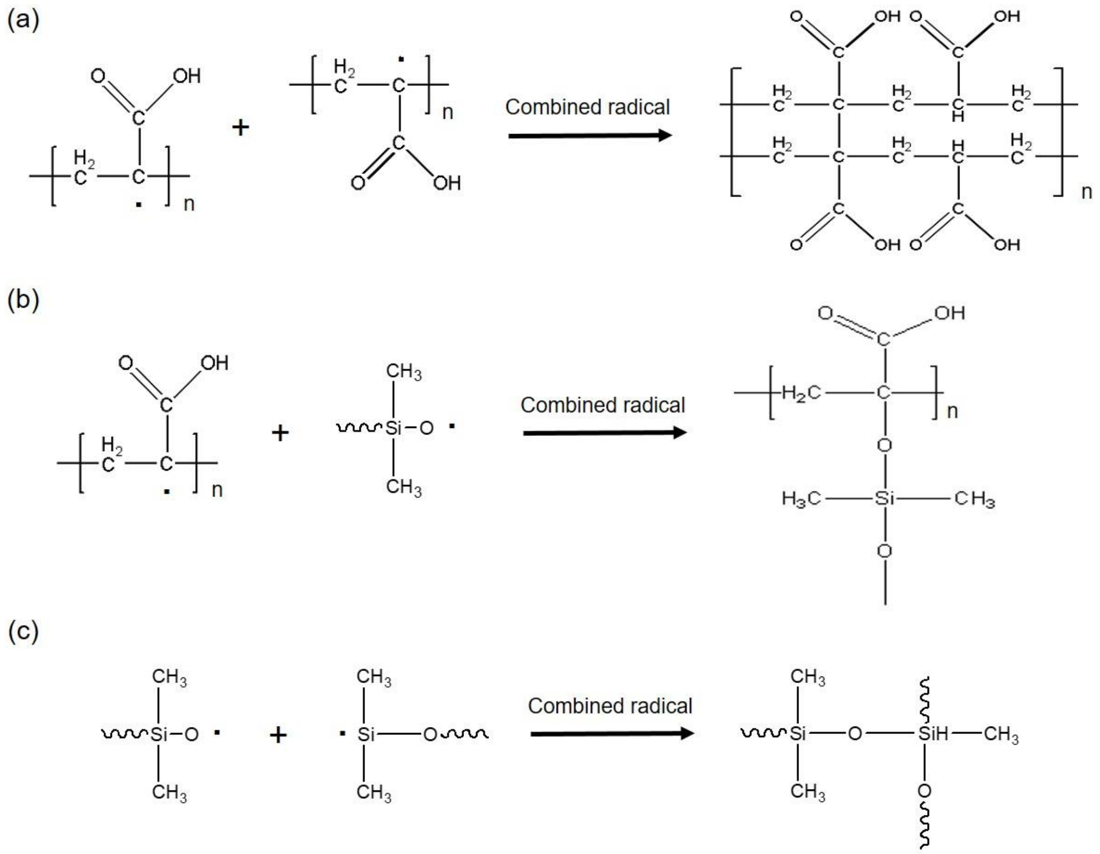

Figure S1. Chemical mechanism for the electron beam cross-linking of PAA-silicone: (**a**) PAA with PAA, (**b**) PAA with silicone, and (**c**) Silicone with silicone.
